# Dataset on the absorption characteristics of extracted phytoplankton pigments

**DOI:** 10.1016/j.dib.2019.103875

**Published:** 2019-03-29

**Authors:** Lesley A. Clementson, Bozena Wojtasiewicz

**Affiliations:** CSIRO Oceans & Atmosphere Hobart TAS 7001, Australia

**Keywords:** Pigments, Phytoplankton, Absorption

## Abstract

This article presents the raw and analysed data on the absorption features of 30 pigments commonly occurring in phytoplankton. All unprocessed absorption spectra are given between 350 and 800 nm. The presented data also gives information on the wavelength of the main absorption peaks together with associated magnitudes of the concentration-specific absorption coefficient.

**Table:** Specifications table

Subject area	Biology
More specific subject area	Phytoplankton biology; Marine biology; Marine bio-optics
Type of data	Tables, figures, separate .txt files with entire measured spectra
How data was acquired	UV-VIS double-beam spectrophotometer (GBC Scientific Equipment Ltd., Cintra 404); software: Cintral ver. 2.2
Data format	Analysed
Experimental factors	Pigments were dissolved in either 100% ethanol or 90% acetone as received from DHI (Denmark) or Sigma Aldrich prior to the measurements being made.
Experimental features	The absorption spectra were measured in a 1-cm quartz-glass cuvette using a dual-beam spectrophotometer against the pure solvent as a blank. The spectra were measured over the 350–800 nm spectral range in 1.3 nm increments.
Data source location	Hobart, TAS, Australia
Data accessibility	All data are provided in this article
Related research article	Baird, M.E., Mongin, M., Rizwi, F., Bay, L.K., Cantin, N.E., Soja-Woźniak, M., Skerratt, J., 2018. A mechanistic model of coral bleaching due to temperature-mediated light-driven reactive oxygen build-up in zooxanthellae. Ecol. Model. 386, 20–37 [1].

**Table:** 

**Value of the data** • This dataset is unique in that it provides the absorption characteristics together with a pigment concentration for 30 different pigments. From this, concentration-specific absorption coefficients are obtained which can be used for both phytoplankton and bio-optical studies. • The dataset can be used in models pertaining to phytoplankton behavior or for theoretical experiments. • The dataset can be used to both compare to in situ experimental results or to help explain experimental results. • The dataset can be base for theoretical experiments in phytoplankton physiology or ecology and marine bio-optics.

## 1 Data

**Fig. 1:**
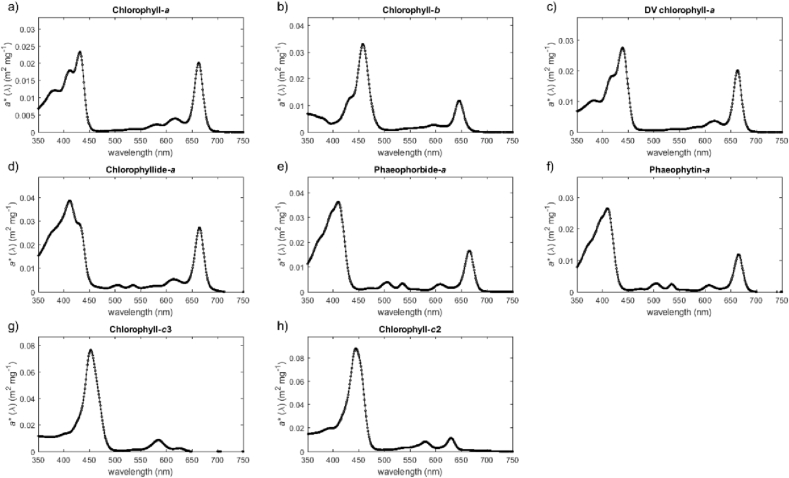
Concentration-specific absorption spectra of (a) chlorophyll-*a*, (b) chlorophyll-*b*, (c) DV chlorophyll-*a*, (d) chlorophyllide-*a*, (e) phaeophorbide-*a*, (f) phaeophytin-*a*, (g) chlorophyll-*c*3, (h) chlorophyll-*c*2.

**Fig. 2:**
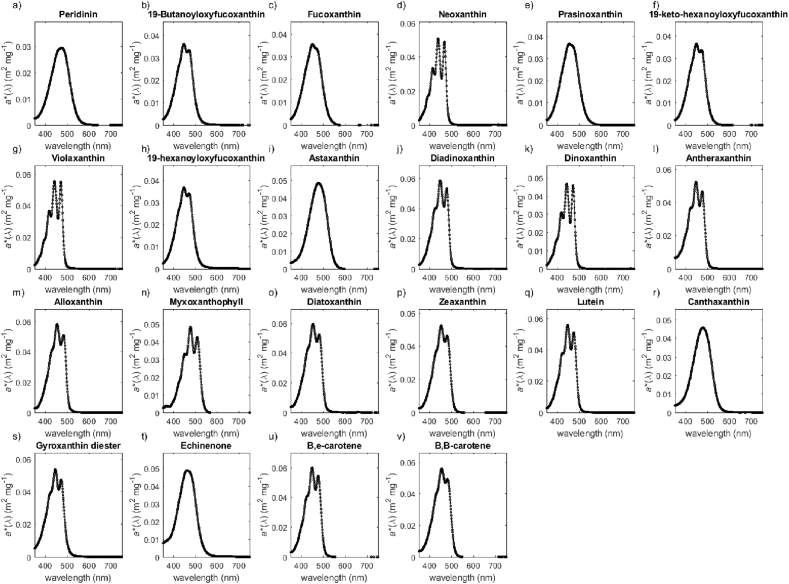
Concentration-specific absorption spectra of (a) peridinin, (b) 19′-butanoyloxyfucoxanthin,(c) fucoxanthin, (d) neoxanthin, (e) prasinoxanthin, (f) 19′-keto-hexanoyloxyfucoxanthin, (g) violaxanthin, (h) 19′- hexanoyloxyfucoxanthin, (i) astaxanthin, (j) diadinoxanthin, (k) dinoxanthin, (l) antheraxanthin, (m) alloxanthin, (n) myxoxanthophyll, (o) diatoxanthin, (p) zeaxanthin, (q) lutein, (r) canthaxanthin, (s) gyroxanthin diester, (t) echinenone, (u) β,ε-carotene, (v) β,β-carotene.

The unprocessed measurement data for the absorption spectra of chlorophylls (chlorophyll-*a*, chlorophyll-*b*, DV chlorophyll-*a*, chlorophyllide-*a*, phaeophorbide-*a*, phaeophytin-*a*, chlorophyll-*c*3, chlorophyll-*c*2) and carotenoids (peridinin, 19′-butanoyloxyfucoxanthin, fucoxanthin, neoxanthin, prasinoxanthin, 19′-keto-hexanoyloxyfucoxanthin, violaxanthin, 19′- hexanoyloxyfucoxanthin, astaxanthin, diadinoxanthin, dinoxanthin, antheraxanthin, alloxanthin, myxoxanthophyll, diatoxanthin, zeaxanthin, lutein, canthaxanthin, gyroxanthin diester, echinenone, β,ε-carotene, β,β-carotene) are given in separate files (Appendix A; carotenoids_concentration_specific_spectra.txt, chlorophylls_concentration_specific_spectra.txt). Fig. 1, Fig. 2 present pigment-specific absorption spectra for each of the analysed pigments and Table 1, Table 2 list the location of the main absorption peaks and the magnitude of the pigment-specific absorption coefficients at these local maxima for chlorophylls and carotenoids, respectively.

**Table 1:** Location of the main absorption peaks and the associated magnitude of the concentration specific absorption coefficient for chlorophyll-*a*, chlorophyll-*b*, DV chlorophyll-*a*, chlorophyllide-*a*, phaeophorbide-*a*, phaeophytin-*a*, chlorophyll-*c*3, and chlorophyll-*c*2.

Name of pigment	Source	Lot/Batch number	Solvent	Main absorption peaks (nm)	Concentration specific absorption coefficient (m^2^ mg^−1^)
Chlorophyll-*a*	Sigma	BCBK2207V	90% acetone	431	0.0233
				663	0.0202
				412	0.0179
				382	0.0122
				617	0.0040
Chlorophyll-*b*	Sigma	SLBF7339V	90% acetone	458	0.0330
				646	0.0118
DV chlorophyll-*a*	DHI	112	90% acetone	439	0.0276
				663	0.0203
Chlorophyllide-*a*	DHI	125	90% acetone	411	0.0387
				665	0.0272
				615	0.0053
				535	0.0029
				506	0.0028
Phaeophorbide-*a*	DHI	105	90% acetone	410	0.0363
				666	0.0166
				505	0.0039
				535	0.0034
				608	0.0031
Phaeophytin-*a*	DHI	107	90% acetone	410	0.0266
				665	0.0119
				505	0.0027
				535	0.0024
				607	0.0021
Chlorophyll-*c*3	DHI	122	90% acetone	452	0.0766
				584	0.0085
				626	0.0024
Chlorophyll-*c*2	DHI	129	90% acetone	444	0.0880
				630	0.0113
				580	0.0083

**Table 2:** Location of the main absorption peaks and the associated magnitude of the concentration-specific absorption coefficient for carotenoids (peridinin, 190-butanoyloxyfucoxanthin, fucoxanthin, neoxanthin, prasinoxanthin, 190-keto-hexanoyloxyfucoxanthin, violaxanthin, 190- hexanoyloxyfucoxanthin, astaxanthin, diadinoxanthin, dinoxanthin, antheraxanthin, alloxanthin, myxoxanthophyll, diatoxanthin, zeaxanthin, lutein, canthaxanthin, gyroxanthin diester, echinenone, b,ε-carotene, b,b-carotene).

Name of pigment	Source	Lot/Batch number	Solvent	Main absorption peaks (nm)	Concentration specific absorption coefficient (m^2^ mg^−1^)
Peridinin	DHI	111	100% ethanol	474	0.0293
19'-Butanoyloxyfucoxanthin	DHI	122	100% ethanol	447	0.0362
				471	0.0335
Fucoxanthin	DHI	119	100% ethanol	449	0.0355
Neoxanthin	DHI	122	100% ethanol	438	0.0508
				466	0.0489
				413	0.0333
Prasinoxanthin	DHI	110	100% ethanol	453	0.0367
19'-keto-hexanoyloxyfucoxanthin	DHI	101	100% ethanol	448	0.0365
				471	0.0337
Violaxanthin	DHI	138	100% ethanol	441	0.0555
				471	0.0552
				417	0.0365
19'-hexanoyloxyfucoxanthin	DHI	116	100% ethanol	446	0.0367
				471	0.0339
Astaxanthin	DHI	105	100% acetone	477	0.0486
Diadinoxanthin	DHI	117	100% ethanol	447	0.0588
				477	0.0535
				426	0.0402
Dinoxanthin	DHI	103	100% ethanol	442	0.0468
				471	0.0458
				417	0.0316
Antheraxanthin	DHI	127	100% ethanol	446	0.0523
				475	0.0464
				423	0.0369
Alloxanthin	DHI	112	100% ethanol	453	0.0583
				482	0.0511
Myxoxanthophyll	DHI	106	100% acetone	477	0.0486
				508	0.0427
				452	0.0333
Diatoxanthin	DHI	133	100% ethanol	452	0.0596
				481	0.0524
Zeaxanthin	DHI	131	100% ethanol	452	0.0524
				479	0.0464
Lutein	DHI	128	100% ethanol	446	0.0559
				474	0.0508
				423	0.0381
Canthaxanthin	DHI	131	100% ethanol	478	0.0458
Gyroxanthin diester	DHI	105	100% ethanol	445	0.0538
				472	0.0473
Echinenone	DHI	121	100% ethanol	461	0.0488
β,ε-carotene	DHI	126	100% acetone	488	0.0600
				476	0.0544
β,β-carotene	DHI	126	100% acetone	454	0.0559
				480	0.0492

## 2 Experimental design, materials, and methods

Pigment standards for chlorophyll-*a* and chlorophyll-*b* were prepared from extracts purchased from Sigma-Aldrich (www.sigmaaldrich.com), while other pigment standards were obtained from DHI (www.dhigroup.com). The source and the batch/lot number of each pigment are given in Table 1, Table 2. The standards were in either 90% acetone, 100% acetone or 100% ethanol (Table 1, Table 2). The final concentrations of the standards were measured by HPLC (High Performance Liquid Chromatography) with the CSIRO method [2], which is a modified version of the [3] technique, using C_8_ column and binary gradient system with an elevated column temperature. Pigments were identified by their retention time and their absorption spectra from the photo-diode array detector. Next, the pigment concentrations were determined through peak integration performed in Empower^©^ software.

The absorption spectra of the pigment standards were measured in a 1-cm quartz-glass cuvette using a Cintra 404 (GBC Scientific Equipment Ltd.) UV-VIS dual-beam spectrophotometer against the pure solvent as a blank. The spectra were measured over the 350–800 nm spectral range in 1.3 nm increments. The absorbance (*OD*) obtained from the measurements was converted to an absorption coefficient (*a*(λ), m^−1^) by multiplying the appropriate baseline-corrected optical density values of each standard by 2.3 and dividing by the optical path length/cuvette thickness (0.01 m):

(1) $$a\left(\lambda \right)=\frac{2.3\;OD\left(\lambda \right)}{0.01}$$

Finally, the concentration specific absorption coefficients (a^*^(λ), m^2^ g^−1^) were calculated by dividing each absorption coefficient by the respective pigment concentration.

Data presented in Fig. 1, Fig. 2 and in Table 1, Table 2 were null-point corrected by subtracting the absorption coefficient value at 750 nm assuming no absorption of pigments in the NIR region of the spectrum [4]. The spectra were also interpolated to yield absorption coefficients between 350 and 750 nm with the resolution of 1 nm using linear interpolation method (MATLAB, *interp1.m*).

Due to differences in the organic solvent and water refractive index (i.e. 1.352 for acetone, 1.361 for ethanol and 1.330 for water), the spectra may be wavelength-adjusted by using the ratio between the refractive index of the solvent and the water as done by [1].
